# Relationship between serum fibroblast growth factor 19 and vascular endothelial growth factor and soluble klotho protein in type 1 diabetic children

**DOI:** 10.1186/s12887-023-03938-x

**Published:** 2023-03-16

**Authors:** Yanjun Zhang, Guimei Li, Feifei Xiao, Bang Wang, Jianchang Li, Xiuhong Jia, Yan Sun, Hongye Chen

**Affiliations:** 1 Department of Pediatric Endocrinology, Shandong Provincial Hospital, Shandong University, 9677 Jingshi Road, Lixia Area, 250021 Jinan, Shandong China; 2 Department of Pediatrics, Bin Zhou Medical University Hospital, 661 Huangheer Road, 256603 Bin cheng Area, Bin Zhou, Shandong China; 3 Department of Pediatric Endocrinology, Shandong Provincial Hospital Affiliated to Shandong First Medical University, 9677 Jingshi Road, Lixia Area, 250021 Jinan, Shandong China

**Keywords:** Type 1 diabetes, FGF19, VEGF, Sklotho, Complication, HbA1c

## Abstract

**Background:**

Fibroblast growth factor 19 (FGF19) takes part in maintaining the balance of glycolipids and may be involved in complications of type 1 diabetes(T1D) in children. This study aimed at at evaluating the relationship among the levels of serum FGF19 and vascular endothelial growth factor(VEGF)and soluble klotho protein(sklotho) in type 1 diabetic children.

**Methods:**

In a cross-section single center study samples were obtained from 96 subjects: 66 T1D and 30 healthy children.Serum FGF19 and VEGF and sklotho concentrations were measured by ELISA. And 66 type 1 diabetes participants were divided into two groups according to T1D duration or three groups according to HbA1c.Furthermore,we compared the serum levels of FGF19 and VEGF and sklotho in different groups.

**Results:**

The concentration of FGF19 was lower in T1D than in the controls(226.52 ± 20.86pg/mu vs.240.08 ± 23.53 pg/L, *p* = 0.03),while sklotho was also lower in T1D than in the controls (2448.67 ± 791.92pg/mL vs. 3083.55 ± 1113.47pg/mL, *p* = 0.011). In contrast, VEGF levels were higher in diabetic patients than in controls (227.95 ± 48.65pg/mL vs. 205.92 ± 28.27 pg/mL, *p* = 0.016). In T1D, FGF19 and VEGF and sklotho was not correlated with the duration of diabetes. FGF19 and VEGF and sklotho were correlated with HbA1c (r=-0.349, *p* = 0.004 and r = 0.302, *p* = 0.014 and r=-0.342, *p* = 0.005, respectively), but not with blood glucose and lipid. Among subjects in the T1D group, concentrations of FGF19,VEGF and sklotho protein were different between different groups according to the degree of HbA1c(P < 0.005).Furthermore, there was a positive correlation between the serum FGF19 concentration and sklotho levels (r = 0.247,*p* = 0.045), and a negative correlation between the serum FGF19 concentration and VEGF level(r=-0.335,P = 0.006). Conclusions: The serum FGF19 levels have a close relation with serum VEGF levels and sklotho levels among T1D subjects. FGF19 may be involved in the development of complications in children with type 1 diabetes through interaction with VEGF and sklotho.

## Background

Type 1 diabetes (T1D) is an autoimmune abnormality featured by beta-cell destruction and insulin deficiency, and it is also a common pediatric chronic disease, although the real prevalence and pathogeny of T1D is unclear [1]. Due to the dependence of exogenous insulin complement on blood glucose control and poor adherence of children, the blood glucose of children with T1D tends to fluctuate greatly and have severe complications, including microangiopathy and cardiovascular disease. Diabetic microvascular lesions can invade patients’ retina, kidney, nerves and other important organs, when serious can cause blindness, kidney failure, neuropathy, and even life-threatening [2–4]. Early diagnosis and treatment of microvascular complications of T1DM are extremely important to improve the quality of life and prolong the life span of patients [5, 6].

FGF19, FGF21 and FGF23 are distinctive in that their capital function as endocrine factors that take part in monitoring important metabolic pathways. FGF19 plays a role in the postprandial inverse feedback regulation of bile acid synthesis and release [7]. FGF19 can lower plasma glucose levels and lower body weight, as well as preventing the development of diabetes [8–10]. It was demonstrated that FGF19 can also reduce hypoxia-reoxygenation-induced cardiomyocyte apoptosis and oxidative stress by inhibiting glycogen synthase Kinase-3β (GSK-3Β) activity and promoting the activation of Nrf2-antioxidant response element [11]. These results suggest that FGF19 has the effects of anti-oxidation and reducing myocardial cell injury induced by Hypoxia. Moreover, serum FGF19 levels are higher in patients with chronic kidney disease than in healthy controls, which may be related to the metabolism of lipids and carbohydrates, and hemodialysis and kidney transplantation can reduce the serum FGF19 level in patients with chronic kidney disease [12].

VEGF acts in promoting endothelial cell proliferation, migration, vasculogenesis, vascular formation, and strengthen vascular permeability. Inhibition of VEGF may help to treat diabetes nephropathy, thus reducing endothelial activation and glomerular inflammation, and finally reversing kidney damage [13]. Similarly, there is a body of evidence that reduced sklotho concentrations may contribute to beta cell apoptosis and the development of type 1 diabetes [14, 15]. Furthermore, the plasma sklotho levels were significantly reduced in children with type 1 diabetes, and their levels were inversely correlated with HbA1c levels, which may suggest a possible involvement in the development of chronic diabetes complications [16]. We therefore hypothesized that there may be interactions between FGF19, VEGF, and sklotho in T1D. To our knowledge, there have been no studies on relationship between FGF19, VEGF and sklotho concentrations in T1D children.

The aim of this study was to evaluate FGF19 ,VEGF and sklotho protein concentration in children with T1D and the correlations of them. We also investigate the correlations of FGF19 ,VEGF, sklotho and classical risk factors of chronic complications of diabetes to provide a better understanding of T1D vascular complications and identify powerful management items for T1D.

## Methods

### Study design and subjects

The study was conducted with inpatients and health examiners from the Shandong Provincial Hospital, Shandong University from Sep 2019 to May 2020. The normal controls were required to be 1–15 years old and to have fasting plasma glucose < 6.1 mmol/L and 2-h plasma glucose (2hPG) < 7.8 mmol/L. The exclusion criteria for all groups were type 2 diabetes, hyperthyroidism or hypothyroidism, severe hepatic disease, chronic renal insufficiency, cancer, acute diabetic complications, and current treatment with systemic corticosteroids. All patients with acute infection presentations or evidence will be excluded. Finally, we recruited 66 participants and 30 healthy children.This study was ratified by the Institutional Review Board of Shandong Provincial Hospital, Shandong University and in conformity with the Declaration of Helsinki.

### Basic data collection

This was a hospital-based prospective observational research. Patients with type 1 diabetes were recruited in the study, while healthy controls were from health check-ups. All participants were questioned and examined by experienced medical workers to complete a questionnaire, which including sex, age, weight, height, disease and medical therapy history. Body mass index (BMI) was calculated by the formula of weight/the height squared.

### Sample collection and laboratory examination

Blood samples were drawn between 07:00 and 09:00 after overnight fasting. Blood samples naturally coagulated for 20 min at room temperature and separated by centrifugation at 2,000 RPM for 20 min within 30 min of blood collection. Serum was carefully collected and then stored in a -80℃ refrigerator until further analysis.

Fasting blood samples were collected to complete laboratory parameters.Serum blood glucose levels, HbA1c concentration, c-peptide level, insulin levels, HDL cholesterol, LDL cholesterol, and triglycerides, total cholesterol, were determined to use standard laboratory procedures.

### Hormonal and biochemical assays

Circulating FGF19 ,VEGF and sklotho levels were detected by a sandwich enzyme immunoassay (Shanghai Enzyme-linked Biotechnology Co., Ltd.,China), following the manufacturer’s instructions. Serum samples for FGF19 ,VEGF and sklotho determination were diluted 1:4 with a dilution buffer prior to the assay. The detection limits were 1.0pg/mL for FGF19 and VEGF, 10 pg/mL for sklotho. Inter-assay and intra-assay ratio of variations were less than 10% and 15% respectively for FGF19 ,VEGF and sklotho.

Serum C-peptide concentrations were detected by commercial RIA kit (Roche Diagnostics GmbH, Mannheim, Germany). Serum insulin levels were detected by commercial ELISA kit (Roche Diagnostics GmbH, Mannheim, Germany). Plasma glucose levels, total cholesterol (TC) and triglycerides concentrations were detected by commercial kit (Beckman Coulter Laboratory Systems Co., Ltd., Suzhou, China). Serum HbA1c concentrations were detected by commercial kit (Dongcao Biotechnology Co., Ltd., Shanghai, China). HDL-cholesterol and LDL-cholesterol concentrations were detected by commercial kit (Weigao Biotechnology Co., Ltd., Weihai, China). The inter-assay and intra-assay variability ratio of all kits was less than 5.0% and 10.0% severally.

The parameters of this study were analyzed using descriptive statistics. The Shapiro-Wilk test is used to detect for normality. In addition, an unpaired t-test was used for the continuous variables which have a normal distribution, and the Mann-Whitney test was used for variables that did not meet the criteria of normal distribution. The one-way analysis of variance (ANOVA) or the Brown-Forsythe Anova test was used for a comparison of more than two groups, with the appropriate post-hoc tests (LSD or Games-Howell multiple comparison test). The relationships between variables were assessed by Pearson Correlation Coefficient or Spearman’s rank correlation coefficient, where appropriate. The data were expressed as an average ± standard deviation and as a percentage of the classified variables. Double tailed p < 0.05 was considered significant. Statistical analysis was performed using the statistical program SPSS (Statistical Package for the Social Sciences Inc. Chicago IL, USA), version 25.

## Results

The study included 66 patients with type 1 diabetes mellitus (50 boys) and 30 children without any evidence of hyperglycemia or any autoimmune disease (controls, 17 boys). None of the studied children had a medical history of retinopathy, arterial hypertension, or cardiovascular disease, and microalbuminuria. All of the participants had normal renal function. The clinical and biochemical characteristics of the study group are shown in Table 1.

**Table 1:** Clinical and biochemical features of type 1 diabetes children

Variable		T1D N = 66		Control group N = 30		P
		Mean(SD) (min, max)		Mean(SD) (min, max)		
Age [years]		7.67(4.26)(1.00,15.00)		8.14(3.27)(2.17,13.25)		0.557
Gender [male %]		56.7%		75.8%		0.059
BMI [kg/m^2^]		17.76(2.95)(13.06,24.68)		17.11(1.83)(14.66,22.44)		0.681
T1D duration [years]		1.57(2.16)(0.04,9.00)		N/A		N/A
HbA1c [%]		9.63(2.47)(5.60,14.9)		5.05(0.60)(4.10,6.10)		< 0.0001
Glucose [mmol/L]		9.40(4.58)(2.92,20.73)		4.71(0.50)(3.9,5.82)		< 0.0001
Totalcholesterol [mmol/L]		4.90(0.91)(2.81,8.17)		4.91(0.71)(3.86,6.13)		0.345
HDL cholesterol [mmol/L]		1.39(0.44)(0.33,2.46)		1.36(0.16(0.94,1.65))		0.703
LDL cholesterol [mmol/L]		2.46(0.91)(0.70,4.81)		2.56(0.74)(0.70,3.75)		0.771
Triglycerides [mmol/L]		1.17(0.47)(0.40,2.44)		1.16(0.43)(0.39,1.83)		0.357
TDD [IU/kg]		0.94(0.23)(0.00,1.42)		N/A		N/A
Basal insulin dose [%of TDD]		33.47(16.13)(6.90,100.00)		N/A		N/A
FGF−19 [pg/mL]		226.52(18.95)(186.82,266.25)		240.08(23.53)(197.20,277.98)		0.03
VEGF [pg/mL]		227.95(48.65)(146.57,376.34)		205.92(28.27)(132.72,249.33)		0.016
sklotho [pg/mL]		2448.67(791.92)(1070.87,3923.97)		3083.55(1113.47)(969.70,5623.69)		0.011

The concentration of FGF19 protein was lower in children with T1D than in the control group (226.52 ± 18.95 pg/mL vs. 240.08 ± 23.53 pg/mL, p = 0.003), and also sklotho protein concentrations in T1D group were lower than in the control group (2448.67 ± 791.92 pg/mL vs. 3083.55 ± 1113.47 pg/mL, p = 0.011). However,the concentration of VEGF protein was higher in children with T1D than in the control group (227.95 ± 48.65 pg/mL vs. 205.92 ± 28.27 pg/mL, p = 0.016).

In T1D patients, the concentrations of FGF19, VEGF and sklotho protein did not differ according to the duration of diabetes (Table 2), and were not interrelated with the duration of diabetes. As shown in Table 2, in the entire cohort of T1D children, FGF19 (228.20 ± 17.55 ng/mL vs. 224.09 ± 20.90 ng/mL), VEGF (230.59 ± 50.54 ng/mL vs. 228.21 ± 42.70 ng/mL) and sklotho protein (2385.09 ± 829.08 ng/mL vs. 2540.51 ± 740.55 ng/mL) concentrations levels were comparable regardless of the duration of the disease.

**Table 2:** Concentrations of FGF19,VEGF and sklotho protein in children with type 1 diabetes according to diabetes duration

Variable		FGF19		VEGF		sklotho
		Mean(SD) (min, max)		Mean(SD) (min, max)		Mean(SD) (min, max)
< 1 years (N = 27)		228.20(17.55)(193.14,266.25)		230.59(50.54)(154.65,376.34)		2385.09(829.08)(1070.87,3923.97)
≥ 1 years (N = 39)		224.09(20.90)(186.82,259.93)		228.21(42.70)(146.57,341.70)		2540.51(740.55)(123.75,3802.56)
*P*		0.391		0.842		0.437

Table 3 gives the correlations between FGF19, VEGF, sklotho protein and selected risk factors. In diabetic patients FGF19, VEGF and sklotho protein concentrations were correlated with HbA1c, however no correlations of FGF19, VEGF and sklotho protein with Glucose and BMI were found. Furthermore, no correlations of FGF19, VEGF and sklotho protein with total cholesterol, HDL-cholesterol, LDL-cholesterol, and triglycerides, were found. FGF19 was positively correlated with sklotho (r = 0.247,P = 0.0.045) and negatively associated with VEGF(r=-0.335, P = 0.006). However, no correlations of sklotho with VEGF were found. VEGF was positively correlated with basal insulin dose(r = 0.339,P = 0.006).

**Table 3:** Spearman rank correlation coefficients between FGF19 protein,VEGF protein,sklotho protein and selected risk factors in children with type 1 diabetes

Variable		FGF19		VEGF		sklotho
		r (P value)		r (P value)		r (P value)
Age [years]		r=−0.227,*P* = 0.067		r = 0.215,*P* = 0.083		r=−0.160,*P* = 0.199
Gender [male %]		r = 0.123,*P* = 0.327		r = 0.040,*P* = 0.750		r = 0.177,*P* = 0.154
T1D duration [years]		r=−0.029,*P* = 0.818		r = 0.095,*P* = 0.449		r = 0.112,*P* = 0.370
BMI [kg/m^2^]		r=−0.063,*P* = 0.613		r = 0.046,*P* = 0.711		r=−0.096,*P* = 0.445
HbA1c [%]		r=−0.349,*P* = 0.004		r = 0.302,*P* = 0.014		r=−0.342,*P* = 0.005
Glucose [mmol/L]		r=−0.128,*P* = 0.307		r=−0.020,*P* = 0.875		r=−0.135,*P* = 0.280
Totalcholesterol [mmol/L]		r = 0.032,*P* = 0.797		r=−0.014,*P* = 0.913		r = 0.176,*P* = 0.158
HDL cholesterol [mmol/L]		r = 0.067,*P* = 0.594		r = 0.091,*P* = 0.467		r = 0.027,*P* = 0.833
LDL cholesterol [mmol/L]		r = 0.142,*P* = 0.256		r=−0.043,*P* = 0.733		r = 0.135,*P* = 0.281
Triglycerides [mmol/L]		r=−0.049,*P* = 0.698		r = 0.046,*P* = 0.715		r=−0.004,*P* = 0.975
TDD [IU/kg]		r=−0.205,*P* = 0.099		r = 0.055,*P* = 0.662		r=−0.026,*P* = 0.835
Basal insulin dose [%of TDD]		r = 0.070,*P* = 0.579		r = 0.339,*P* = 0.006		r=−0.105,*P* = 0.405
FGF−19 [pg/mL]		-		r=−0.335,*P* = 0.006		r = 0.247,*P* = 0.045
VEGF [pg/mL]		r=−0.335,*P* = 0.006		-		r = 0.000,*P* = 0.999
sklotho [pg/mL]		r = 0.247,*P* = 0.0.045		r = 0.000,*P* = 0.999		-

**Fig. 1:**
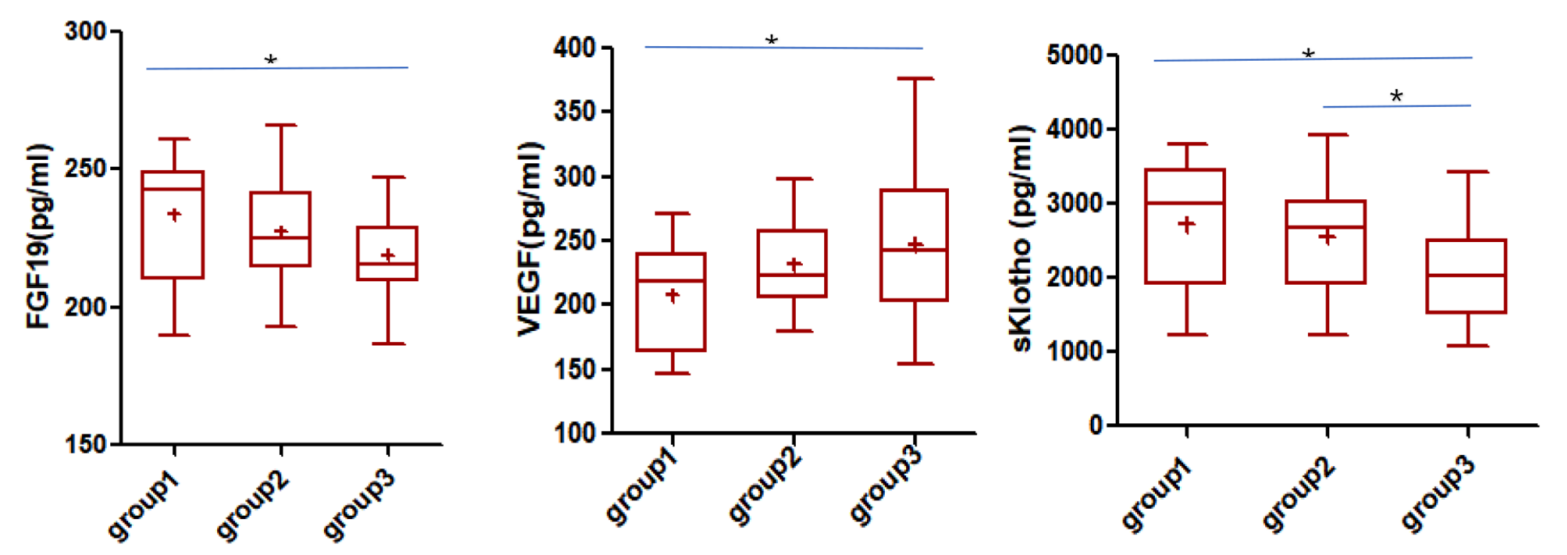
Concentrations of FGF19, VEGF and sklotho protein in children with type 1diabetes according to the HbA1c levels degree (G1(group1):HbA1c < 7.5%, G2(group2):HbA1c 7.5-11.0%, G3(group3): HbA1c > 11.0%; * marks statistically significantly variables)

To evaluate if the concentrations of FGF19, VEGF and sklotho correspond to the degree of metabolic control, the group of T1D patients was divided according to the levels of HbA1c: HbA1c < 7.5% (group 1, N = 19), HbA1c 7.5-11.0% (group2, N = 26) and HbA1c > 11.0% (group3, N = 21). Significant differences of the concentrations of FGF19, VEGF and sklotho, depending on the HbA1c levels were found(p = 0.045, p = 0.039, and p = 0.018, respectively). Further analyzes showed that the concentration of FGF19 and sklotho in the group of patients with the highest HbA1c levels was significantly lower compared to the group of patients with lower HbA1c values, contrariwise, the concentration of VEGF in the group of patients with the highest HbA1c levels was higher than that in the lower HbA1c levels (Table 4; Fig. 1).

**Table 4:** Concentrations of FGF19,VEGF and sklotho protein in children with type 1 diabetes according to the degree of metabolic control

Variable		FGF19		VEGF		sklotho
		Mean(SD) (min, max)		Mean(SD) (min, max)		Mean(SD) (min, max)
G1:HbA1c < 7.5% (N = 19)		233.71(21.37)(189.53,260.83)		207.58(40.32)(146.57,271.27)		2723.73(820.03)(1232.75,3802.56)
G2:HbA1c 7.5−11.0% (N = 26)		227.33(18.33)(193.14,266.25)		231.61(32.64)(180.06,298.98)		2559.68(770.88)(1232.75,3923.97)
G3:HbA1c > 11.0% (N = 21)		210.01(15.01)(186.82,247.29)		247.08(60.39)(154.65,376.34)		2062.37(667.53)(1070.87,3418.10)
*P*		0.045		0.039		0.018
*P(post-hoc analysis)*		G1 vs. G3 p = 0.014		G1 vs. G3 p = 0.049		G1 vs. G3 p = 0.007 G2 vs. G3 p = 0.028

## Discussion

Our results suggest that children with type 1 diabetes have significantly lower FGF19 and soluble klotho concentrations and higher VEGF concentrations compared to healthy individuals, especially in individuals with the worst metabolic control, being expressed as high HbA1c levels. Furthermore, FGF19 was positively related with sklotho levels, and negatively related with VEGF levels. There was no association with other traditional risk factors for chronic complications of diabetes, such as disease duration, and BMI. To the best of our knowledge, this is the first study focusing on the correlation of FGF19, VEGF, and sklotho levels in T1D.

T1D is characterized by destruction of islet β cells, which leads to insulin deficiency and glucose metabolic disorders [17, 18]. T1D starts early, mostly in children and adolescents, with poor self-management ability, poor glycemic control, and a high incidence of various acute and chronic complications. Early diagnosis and treatment of microvascular complications of T1D are extremely important to improve the quality of life and prolong the life span of patients.

Serum FGF19 levels were positively correlated with glucose metabolic efficacy and negatively correlated with hepatic glucose production, and this effect was insulin-independent, and reduced FGF19 levels promoted elevated fasting glucose [19, 20]. However, the relationship between FGF19 and T1D remains unclear. In this study, we found that serum FGF19 levels were lower in T1D patients with duration from less than 1 month to 9 years than in healthy controls. Consistent with our studies, previous studies found decreased serum fibroblast growth factor 19 level was a risk factor for type 1 diabetes newly diagnosed T1D patients [21]. The glycemic-lowering functions of FGF19 are non-insulin-dependent manner [22, 23]. Previous studies have also found that FGF19 is associated with lipid metabolism in patients with metabolic syndrome and healthy controls [24–26]. In the present study, we did not find a significant relevance between FGF19 and lipid index. One possible explanation was that the sample size was insufficient. Therefore, more samples will be recruited to determine the correlation between FGF19 and lipid in the future study.

The relationship of FGF19 with diabetic complications was unknown. Jingyi Hu et al. found that serum FGF19 level could help to predict the development of atherosclerosis in men with type 2 diabetes [27]. FGF19 showed better mitochondrial efficiency, which may be related to higher cardiac contractility in the diabetic heart, and the modulation of PGC-1a, responsible for FGF19 activation, may be a therapeutic target for diabetic cardiomyopathy [28].

VEGF was associated with vascular endothelial injury in diabetes. Hyperglycemia changes the retinal microenvironment, resulting in abnormal endothelial cell function caused by hypoxia and increased VEGF expression [29]. Serum VEGF in type 2 diabetes patients were significantly higher than those in the control group, and serum VEGF may be involved in the diabetic kidney disease (DKD) process through inflammation, angiogenesis, and endothelial injury [30]. Elevated serum VEGF levels in adult patients with T1D may reflect early microcirculation disorders in the course of diabetes mellitus [31]. Our results showed that, compared with the control group, serum levels of VEGF were significantly elevated in children with type 1 diabetes and increased as HbA1c protein increased. VEGF and FGF cooperate in angiogenesis in vitro and in vivo [32, 33].VEGF is synergistic with FGF19 in neoplastic disease [34]. We hypothesized that there were some associations between serum FGF19 and VEGF in children with type 1 diabetes, but the relationship between them was unclear. In our study, correlation analysis showed that serum VEGF was negatively correlated with FGF19. The results suggest that FGF19 may play a role in reversing diabetic microangiopathy through VEGF.

Meanwhile, we also found that the basal insulin dose was positively correlated with VEGF (r = 0.339,*P* = 0.006). Likewise insulin upregulated VEGF expression in diabetic ovary [35]. Similarly, anti-VEGF treatment increased insulin sensitivity in both young and aged mice [36]. Therefore, according to our study, we concluded that, there were some associations between VEGF and insulin in the internal environment of hyperglycemia in diabetes patients.

Klotho is a transmembrane protein mainly expressed in renal tubules. Soluble Klotho is the result of proteolytic cleavage of the extracellular domain of transmembrane Klotho, or is the product of alternative splicing of the Klotho gene. The Klotho protein is involved in the phosphate metabolic concentrations together with the fibroblast growth factor. Zubkiewicz-Kucharska A et al. reported a significant reduction in sklotho levels in children with type 1 diabetes, associated with HbA1c and HDL cholesterol, but not with adhesion molecule concentration and duration of disease, and the level of HbA1c was inversely associated with Soluble Klotho, suggesting a possible involvement in the development of chronic diabetic complications [16]. Similar to our findings, the serum level of sklotho was reduced in children with type 1 diabetes mellitus, and the degree of the reduction was positively correlated with HbA1c. FGF19 binds to the tyrosine kinase receptor FGFR3c or FGFR4 to mediate its function, and it must bind to the transmembrane glycoprotein Klotho gene family in order to play the regulatory role of endocrine hormones [37, 38]. β-Klotho is necessary for the activity of FGF19 and FGF21 factors [39]. So we assumed that there were some associations between serum FGF19 and sklotho in children with type 1 diabetes. Our results suggested a positive relationship between FGF19 and sklotho (r = 0.247,*p* = 0.045) and maybe there were some mechanisms linking FGF9 and kolotho.

Expression of VEGF was elevated in endothelial cells after exposure to uremic serum from end-stage renal disease patients with diabetes and/or hypertension, and this damage activated the initiation of vascular repair processes in these cells by increasing the expression of VEGF [40]. So the increased levels of VEGF may be a protective response of the organism. In our results, FGF19 is negatively correlated with the expression level of VEGF, and this relationship in vascular endothelial injury maybe was a complementary relationship.The pathophysiologic relevance of these findings remains to be elucidated.

## Conclusion

This study reports the significantly lower levels of FGF19 and sklotho in children with type 1 diabetes, and higher level of VEGF, correlated with HbA1c, but not with the lipid concentrations nor the duration of the disease. Negative correlation between the levels of FGF19 and VEGF, and positive correlation between the levels of FGF19 and Soluble Klotho may suggest its possible involvement in the development of chronic complications of diabetes.

## Data Availability

The datasets used and/or analyzed during the current study are available from the corresponding author on reasonable request.

## Abbreviations

FGF19: Fibroblast growth factor 19
VEGF: vascular endothelial growth factor
sklotho: soluble klotho protein
T1D: Type 1 diabetes
